# Delivery of average assured pressure support (AVAPS) through tracheostomy in paediatric patients

**DOI:** 10.1002/rcr2.1269

**Published:** 2023-12-09

**Authors:** Vishal Saddi, Antonia O'Connor, Ganesh Thambipillay, Arthur Teng

**Affiliations:** ^1^ Department of Sleep Medicine Sydney Children's Hospital Sydney New South Wales Australia; ^2^ School of Women and Children's Health, Faculty of Medicine University of New South Wales Sydney New South Wales Australia

**Keywords:** average volume‐assured pressure support, paediatrics, tracheostomy

## Abstract

Average volume‐assured pressure support (AVAPS) mode has been available since 2009 and allows the ventilator to deliver a constant pre‐set tidal volume by automatically adjusting the inspiratory pressures within a set range. Data in AVAPS mode use is limited in both paediatric populations, and in patients who are ventilated through a tracheostomy. This case series reports on the successful use of AVAPS mode in four paediatric patients with tracheostomy ventilation.

## Graphical abstract

Average volume assured pressure support (AVAPS) mode enables the ventilator to deliver a constant pre‐set tidal volume by automatically adjusting the inspiratory pressures within a set range. Although AVAPS mode has been available since 2009, data on its use in children are limited. We report safe and successful use of the AVAPS mode in four paediatric patients with tracheostomy ventilation.

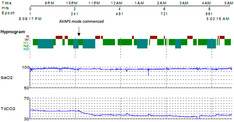

## INTRODUCTION

Average volume assured pressure support (AVAPS) mode enables the ventilator to deliver a constant pre‐set tidal volume by automatically adjusting the inspiratory pressures within a set range. Although AVAPS mode has been available since 2009, data on its use in children are limited. The authors recently published their experience with the non‐invasive use of AVAPS in a cohort of children resulting in improved gas exchange and hypoventilation compared to use of conventional bilevel positive airway pressure (BPAP).^1^ Data on the use of AVAPS through a tracheostomy are lacking. We report safe and successful use of the AVAPS mode in four paediatric patients with tracheostomy ventilation.

## CASE SERIES

### Case 1

**FIGURE 1:**
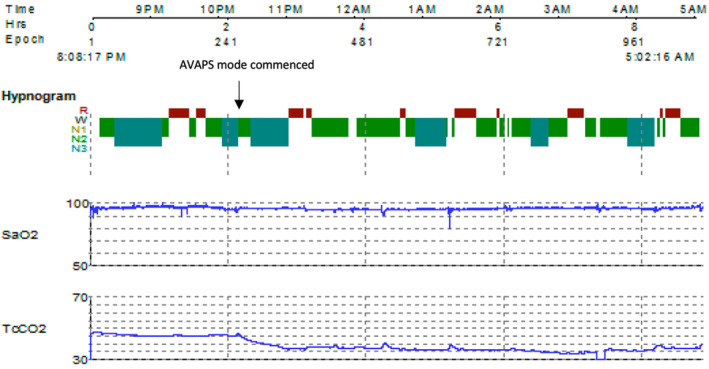
Sleep study summary from Case 1—note improvement in TcCO2 with AVAPS mode commencement.

A 14‐year‐old with Prader–Willi syndrome and a background of non‐accidental traumatic brain injury with severe developmental delay was referred to our facility at a young age for monitoring for sleep disordered breathing. He was commenced on BPAP for evidence of obstructive and central hypoventilation at 7 years of age. However, adherence remained unsatisfactory. He had multiple presentations to the hospital with lower respiratory tract infection and respiratory failure. Eventually, a tracheostomy was performed for a secure airway. Ventilation was commenced on conventional BPAP (IPAP 18 cm H_2_O, EPAP 5 cm H_2_O, back up rate 20 breaths per minute, inspiratory time 1.2 s, rise time 1) through a tracheostomy. There was improvement in gas exchange however, PCO2 on venous gases remained elevated to 60 mmHg. To improve hypoventilation, AVAPS was trialled through tracheostomy. The gas exchange recording from the sleep study are depicted in Figure 1.

### Case 2

A 9‐year‐old boy with congenital central hypoventilation syndrome (CCHS) was managed by our institute since his initial presentation at 2 years of age with a hypoglycaemic seizure and severe hypoventilation. Subsequent genetic testing revealed PHOX2B mutation. A tracheostomy was performed to ensure a secure airway. Conventional BPAP was trialled through tracheostomy at pressures of IPAP 16 cm H_2_O, EPAP 4 cm H_2_O, back up rate 28 breaths per minute, inspiratory time 0.8 s. On these settings, transcutaneous CO2 was persistently elevated greater than 50 mmHg to a maximum of 70.2 mmHg. AVAPS was trailed and achieved improved ventilation, with transcutaneous CO2 remaining less than 50 mmHg to a maximum of 49 mmHg.

### Case 3

A 6‐year‐old boy with a background of Gullian–Barre syndrome and tracheostomy ventilation was commenced on AVAPS mode. His ventilatory status remains stable on the AVAPS mode through a tracheostomy for a period of 3 years now.

### Case 4

An 8‐year‐old boy with a background of Moebius type syndrome with severe congenital central hypotonia, who also had a tracheostomy for left vocal cord palsy, laryngomalacia and bulbar dysfunction was managed by our facility for hypoventilation. He was trialled on AVAPS mode due to difficulties with sleep maintenance and comfort. He tolerated this well with good gas exchange.

## DISCUSSION

Although several paediatric case reports and case series have demonstrated efficacy of AVAPS mode used non‐invasively,^2^, ^3^, ^4^, ^5^ the use of AVAPS mode to provide effective and safe ventilation through the tracheostomy in children has been described in only one recent case series.^6^ AVAPS offers several advantages over conventional BPAP. Fixed pressure BPAP is unable to compensate for changes in airway resistance, respiratory system compliance or sleep stage‐related respiratory effort that can adversely impact on tidal volume delivery. During REM sleep, breathing is mainly diaphragmatic owing to physiologic inhibition of intercostal muscles accompanied by decreases in tidal volume. These effects are exaggerated in patients with underlying respiratory issues/disease leading to inconsistent ventilation during sleep. In contrast to fixed pressure BPAP, the AVAPS mode aims to provide a more consistent average level of ventilation by using an internal algorithm to automatically adjust the inspiratory pressure support within pre‐set limits to achieve a clinician‐determined average tidal volume target.

In our cases, AVAPS was delivered through the tracheostomy using recommendations similar to its use in patients ventilated non‐invasively. Ideal body weight was calculated for all cases. The recommended range of a tidal volume of 6–10 mL/kg of ideal body weight was used.^7^ Ventilation was delivered using a Philips Trilogy 100 device through a single passive circuit with an exhalation port. Humidification was provided in all cases using a Fisher and Paykel HR850 humidifier. Minimum and maximum inspiratory pressures were selected, an expiratory pressure, back up respiratory rate and rise time. In all cases, air leak was acceptable. Although one study,^8^ using a lung bench raised concerns about the ability of single limb ventilators to maintain the pre‐set tidal volume during periods of unintentional leak, we did not notice significant variation in set and delivered tidal volumes. It must be emphasized that the auto EPAP titrating feature on AVAPS called AVAPS‐AE is not recommended for invasive ventilation.

Compared with the more conventional modes for ventilation delivered invasively, AVAPS is limited in its ability to be delivered to younger infants. This is due to the minimum tidal volume threshold of 50 mL and high trigger sensitivity limits. In addition, there is a paucity of data of the use of the AVAPS mode in tracheostomy patents. Other issues such as leak compromising ventilation are not isolated to those receiving invasive ventilation via the AVAPS mode and is a potential problem in all those receiving positive pressure ventilation via the tracheostomy.

In summary, our case reports the successful use of the AVAPS mode in four paediatric patients with tracheostomy. The cases highlight that the AVAPS mode may be safe and effective when delivered invasively through a tracheostomy tube. Prospective, longitudinal studies are needed to evaluate the benefits of invasive ventilation using the AVAPS mode.

## AUTHOR CONTRIBUTIONS

**Vishal Saddi**: Responsible for conceptualization of work; drafting and preparing the original manuscript; editing the manuscript. **Antonia O'Connor**: Responsible for drafting and preparing the manuscript; editing the manuscript. **Ganesh Thambipillay**: Responsible for conceptualization of work; editing the manuscript and supervision. **Arthur Teng**: Responsible for conceptualization of work; editing the manuscript and supervision.

## CONFLICT OF INTEREST STATEMENT

None declared.

## ETHICS STATEMENT

The authors declare that appropriate informed consent was obtained for the publication of this manuscript and accompanying images.

## Data Availability

Data sharing not applicable to this article as no datasets were generated or analysed during the current study.
